# “It’s okay to not know …” a qualitative exploration of faculty approaches to working with uncertainty

**DOI:** 10.1186/s12909-022-03180-6

**Published:** 2022-03-01

**Authors:** Jenny Moffett, Elizabeth Armitage-Chan, Jennifer Hammond, Síle Kelly, Teresa Pawlikowska

**Affiliations:** 1 Health Professions’ Education Centre, RCSI University of Medicine and Health Sciences, 123 St. Stephen’s Green, Dublin 2, D02 YN77 Ireland; 2 RVC, Hawkshead Ln, Brookman’s Park, Hatfield, AL9 7TA England; 3 University of Glasgow, Garscube Estate, 464 Bearsden Road, Glasgow, G61 1QH Scotland; 4 RCSI, Smurfit Building, Beaumont Hospital, Beaumont Road, Dublin 9, D09 YD60 Ireland

**Keywords:** Ambiguity, Attributes, Faculty development, Taxonomy, Uncertainty, Undergraduate

## Abstract

**Background:**

Whilst it is recognised that a capacity to manage uncertainty is an essential aspect of working as a healthcare professional, there is little clear guidance on how to facilitate student learning in this domain. A lack of faculty development opportunities also suggests that health professions’ educators may feel ill-equipped to assist students in developing effective approaches to uncertainty. The purpose of this study was to explore a faculty development intervention designed to help educators unpack students’ experiences of uncertainty, and identify attributes which may help students to manage uncertain situations.

**Methods:**

This qualitative study was informed by a constructivist methodological approach, where participants were encouraged to share meaning around the nature of uncertainty in health professions’ education. Two 90-min faculty development sessions were held. These sessions invited participants to apply Han *et al*.’s taxonomy of uncertainty to role-played scenarios of student uncertainty within a focus group setting. Focus group data were collected, and examined using a two-stage, hybrid approach of deductive and inductive thematic analysis.

**Results:**

Han *et al.*’s taxonomy helped participants to identify multiple sources and issues of uncertainty in the role played scenarios, thus unveiling the extent of uncertainties encountered by health professions’ learners. Data analysis revealed four themes overall: “Sources of uncertainty”, “Issues of uncertainty”, “Uncertainty attributes”, and “Learning environment.” Participants also contributed to a list of attributes which they considered helpful to undergraduate health professions’ students in managing uncertain situations. These included an awareness of the nature of uncertainty within healthcare practice, an ability to recognise uncertainty, and adopting attitudes of adaptability, positivity, and resilience.

**Conclusions:**

This study highlights the successful use of Han *et al.*’s taxonomy of uncertainty within a faculty development setting. Our findings suggest that the taxonomy is a practical and versatile tool that health professions’ educators can use in shared reflections and conversations around uncertainty with students or colleagues.

## Background

Health professionals encounter uncertainty on a daily basis, as they attempt to make sense of complex situations and make decisions despite limited or unclear information. A capacity to manage uncertainty is essential for the wellbeing of health professionals [1, 2] and the patients in their care [3]. There have been persistent calls to address uncertainty within health professions’ curricula [4–7], thus preparing our graduates of the future for a “supercomplex” world [8].

However, whilst a capacity to manage uncertainty has been recognised as a core dimension of professional competence [9], and is now a regular addition to professional competency-based frameworks [10–13], there is no consensus on how to support effective learning around uncertainty. A recent scoping review reveals that although health professions’ students meet uncertainty regularly within the context of their undergraduate training, they appear to receive little, if any, formal training on how to manage this [14].

Uncertainty can be understood as a “subjective perception of not knowing what to think or what to do” [15]. This is a frequent experience for health professionals which can influence clinical decision-making and professional practice. Yet, there are few published examples of teaching interventions which specifically address uncertainty management. One explanation may be the lack of clear evidence which links training around uncertainty to explicit, measurable and positive outcomes. White and Williams [16] state that although there is a “substantial body of evidence in support of the implementation of formal teaching regarding uncertainty... There have been no trials on which to base judgements about the long-term effectiveness, outputs, value for money and beneficial effects on practitioner resilience and performance [of this teaching].”

This situation may be about to change. Although research into uncertainty in health professions’ education has existed for more than half a century [17], more recent work has built a compelling case for using educational interventions to influence learners’ uncertainty management [18–20]. Stephens *et al*. [21] state that “education may be a formidable moderator of tolerance of uncertainty, with multiple aspects of the learning environment impacting student tolerance of uncertainty. Therefore, educators should feel confident in trying to incorporate tolerance of uncertainty paradigms into existing curricula, even traditionally content-heavy science courses.” Returning to our opening question around teaching interventions, perhaps the word “should” holds a clue? Although educators *should* feel confident to offer teaching around uncertainty, it is distinctly possible that they do not.

A recent increase in research around uncertainty in health professions’ education has also revealed subtle but important shifts in thinking [14]. Whilst older studies have focused on how to raise learners’ tolerance for ambiguity, newer work hints at a more nuanced balance between tolerance and intolerance [22]. In other words, educators face ambiguity in planning how to help their students to face ambiguity. Further to this, educators may also view uncertainty, overall, with some trepidation. As Hillen *et al.* [23] explain: “Uncertainty can be aversive; large bodies of research from multiple disciplines, both in and outside of the health care domain, have demonstrated that uncertainty provokes fear, worry and anxiety, perceptions of vulnerability, and avoidance of decision-making.” These factors combine to explain why educators may not feel confident to offer teaching around uncertainty or, indeed, to disclose the full extent or nature of their own uncertainties.

Despite these many potential reasons for health professions’ educators’ reluctance to engage with uncertainty, it seems clear that they act as an influential presence in helping learners to cope with uncertain situations, e.g. through role modeling and mentoring [14, 24]. It is likely, then, that educators may need assistance to reach their potential here, making the ways that they themselves respond to uncertainty explicit and tangible as they guide students. As Domen [25] states, “a greater emphasis should be placed on the teaching of ambiguity to residents and faculty who, ultimately, have the greatest influence on the qualities and behaviors we hope to instill in our students, residents, and other learners.” There is, however, a surprising lack of research into faculty development around strategies for uncertainty [26, 27], and little guidance on how to equip faculty to recognise and engage with students’ experiences of uncertainty. It is reasonable to consider that effective faculty development interventions may empower health professions’ educators to notice and harness opportunities to support learning, both formal and informal, around uncertainty.

The purpose of this study is to explore a faculty development intervention designed to help educators unpack simulated experiences of student uncertainty. Using role-played vignettes derived from real-life experiences to trigger discussions around uncertainty, we aimed to deepen educators’ understanding of where uncertainty manifests within health professions’ education, and how students can be helped to manage its accompanying challenges. With regards to the latter, we wanted to gather educators’ perspectives on the student attributes (knowledge, skills and attitudes) that would represent foundational competence in managing uncertainty [28]. By specifically defining the attributes that make up the construct of uncertainty management and tolerance, we hoped this would make both implicit and tacit learning around uncertainty more explicit, as well as providing educators with a framework for supporting students when they reflect on uncertain situations.

**Fig. 1:**
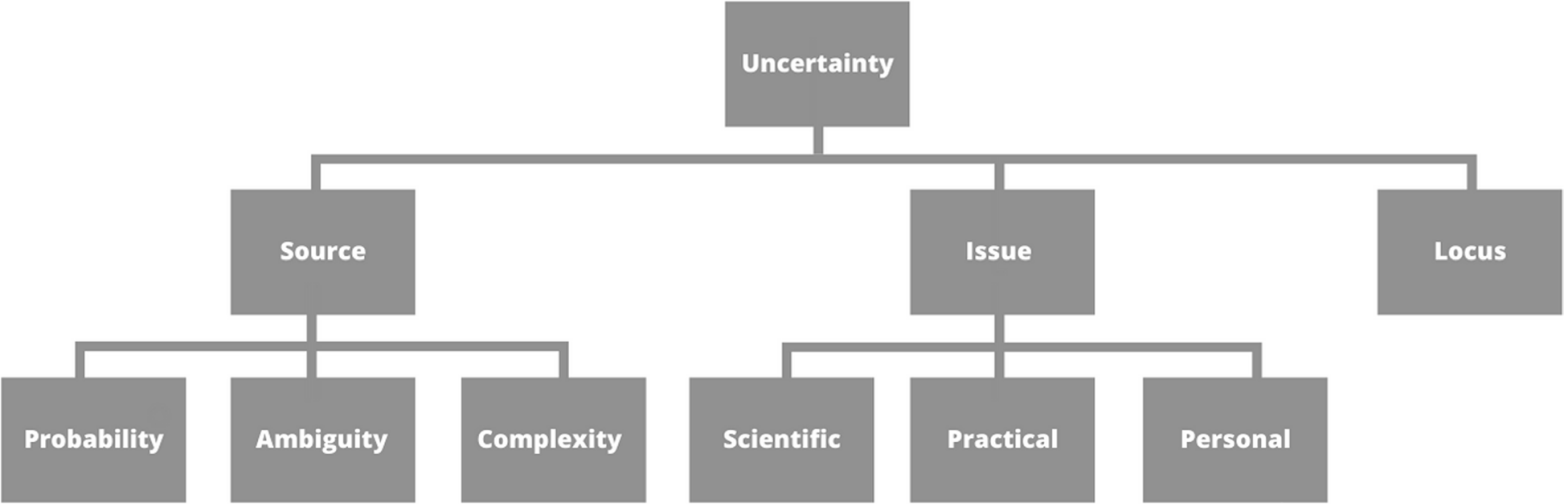
Han and colleagues’ (2011) taxonomy organises uncertainty according to three dimensions: sources, issues and locus

Our faculty development intervention employed Han and colleagues’ [29] taxonomy of uncertainty as a conceptual framework. This taxonomy organises experiences of uncertainty according to three dimensions: source, issues and locus (Fig. 1). The *locus* of the uncertainty is the person to which the uncertainty relates. The *sources* of uncertainty are where the uncertainties arise from. These can be categorised as probability (the indeterminacy of a future event occurring), ambiguity (the lack of adequate, reliable or credible information) or complexity (aspects of the situation that make it difficult to understand). The *issues* of uncertainty are the substantive issues about which an individual is uncertain. These can be categorised as scientific or data-centred (uncertainty related to a medical condition), practical (uncertainty related to the system, structures, or processes) or personal (uncertainty related to the individual). We chose this taxonomy as it has been demonstrated as a practical way to facilitate “an organized approach to the problem of uncertainty” [29] in a wide range of healthcare contexts [30–36]. Using this taxonomy also allowed us to situate our study within the wider body of literature on uncertainty in healthcare, helping to contribute to “a more systematic program of research based upon shared, integrative conceptual models” [37].

Here, we used the taxonomy to help faculty participants to “distinguish and understand … specific uncertainties” [33], and reflect on how uncertainty arises and unfolds in health professions’ education. Through applying the taxonomy to role-played scenarios, we reasoned that participants would gain deeper insight into what attributes could help undergraduate students to manage uncertain situations. Our specific research questions were:

- “In what ways does the use of Han *et al*.’s taxonomy of uncertainty support health professions educators’ understanding of this concept when used experientially to support faculty development?”; and,
- “What do health professions’ educators consider as key attributes for undergraduate learners with respect to managing uncertainty?”

To the researchers’ knowledge, it is the first time that that this conceptual framework has been used in an educational setting to generate a deeper understanding of uncertainty in a context of faculty development.

## Methods

### Study design

We used a qualitative study design which was informed by a constructivist methodological approach, where understanding about uncertainty was co-constructed by participants, using simulated scenarios as triggers for conversation and exploration of this concept [38]. This study is reported in accordance with O’Brien *et al*.’s [39] Standards for Reporting Qualitative Research.

### Intervention

The faculty development session was underpinned by a social constructivist theory of learning. Using this approach, participants were encouraged to share meaning around the nature of uncertainty in health professions’ education. The specific instructional design of the intervention was inspired by Armitage-Chan and Whiting’s [40] use of role-play to provoke faculty discussion around student professionalism learning outcomes, with an overall objective of helping attendees to “practice, reflect on, and develop important skills in a predictable and safe learning environment”.

Each session was 90 min in length and attendees were notified of two learning outcomes, i.e., that, by the end of the session, they would be able to (i) analyse uncertain situations using a recognised taxonomy of uncertainty, and (ii) identify attributes that could help prepare undergraduate health professions’ students to navigate uncertain situations. There were three components to each session: opening plenary (30 mins); focus group work (30 mins); and final whole group work (30 mins).

In the opening plenary, JM addressed contemporary definitions of uncertainty and highlighted early findings from a scoping review which explored how undergraduate health professions’ students learned to engage with uncertainty which had been carried out by three members of the research team (JM, JH and TP) [14]. This was followed by a description of Han *et al*.’s taxonomy of uncertainty [29].

Next, the participants were invited to engage in a focus group activity. Participants had been pre-assigned to a group by facilitators according to profession, so that each focus group had a multi-professional profile. Group sizes ranged from four to six participants. In these focus groups, participants were presented with two role-played scenarios which they observed and discussed in sequence.

The role plays were scripted using content from first-hand student accounts of uncertainty documented in the literature [41–43],and enacted by experienced facilitators (Table 1). A live role play was used during the face-to-face focus group, and a pre-recorded video role play was used during the online focus group.

**Table 1:** Uncertainty role play scenarios

**Scenario 1**	
**This conversation takes place in a quiet room off a ward in a busy teaching hospital. A clinical educator meets with final-year medical student Alice (right), who is on a general medicine rotation. Alice has just returned from a difficult interview with an elderly patient who was admitted to the ward with symptoms of pneumonia.** *(Adapted from: Steinauer, J. E., O’Sullivan, P., Preskill, F., ten Cate, O., & Teherani, A. (2018). What Makes “Difficult Patients” Difficult for Medical Students?. Academic Medicine, 93(9), 1359-1366.)* Available online at: https://rcsi.cloud.panopto.eu/Panopto/Pages/Viewer.aspx?id=bf0c2b6a-d8fb-4067-8373-aba600a86cfa	
**Scenario 2**	
**This conversation takes place in a small group teaching room of a university. Two first-year nursing students are attending a problem-based learning (PBL) session that takes place during a module on infectious disease prevention. The guidelines and facilities provided for the activity are less than optimal, and Dena (right) is confused.** *(Adapted from: Biley, F. and Smith, K. (1999). Making sense of problem-based learning: the perceptions and experiences of undergraduate nursing students. Journal of Advanced Nursing, 30(5), pp.1205-1212.)* Available online at: https://rcsi.cloud.panopto.eu/Panopto/Pages/Viewer.aspx?id=72807b43-baa6-4e56-84c0-aba600a84cad	

In the first scenario, participants watched “Alice”, a final-year medical student, disclose her uncertainty when, during her hospital placement, her patient’s unexpected communication and behaviour made her feel uncomfortable, limiting her capacity for clinical history-taking. The role play captures her debrief with a clinical tutor. Participants were asked to analyse Alice’s experience using Han *et al*.’s taxonomy as a guiding framework, i.e., “If you place Alice at the “locus” of uncertainty here, what sources and issues of uncertainty existed for her?” The participants were then asked: “What knowledge, skills or attitudes might have helped Alice to manage uncertainty in this situation?”

In the second scenario, participants watched “Dena”, a first-year nursing student, discuss her uncertainties around a poorly organised problem-based learning (PBL) session with a fellow student. Again, participants were asked to apply Han *et al*.’s taxonomy, this time with Dena at the locus of uncertainty. They were also asked to consider what knowledge, skills or attitudes might have helped her to manage uncertainty.

After both scenarios had been observed and discussed, participants were invited to reconvene as a large group. Participants in the group were then asked to contribute to a list of knowledge, skills and attributes that could help undergraduate health professions’ students to prepare for uncertainty more generally.

### Participants

The faculty development intervention was offered on two occasions, with two distinct recruitments of participants. The first session was offered as part of the Irish Network of Healthcare Educators’ annual conference [44]. The session was promoted to all conference attendees through the conference website and programme. The second session was offered to members of staff at RCSI, a health professions-specific university with an international campus (RCSI, 2000). This online session was promoted through email lists and the university’s in-house social media platform (WorkVivo; Cork, Ireland), and was facilitated using Zoom web-conferencing software (Zoom; San José, USA). Participants in both sessions were informed that they could take part without being included in the research study. To qualify for inclusion in the study, participants needed to have an active role in supporting learning for health professions’ students, and to have provided consent for their comments to be captured. There were no specific exclusion criteria. No incentives were offered to take part in this study.

The faculty development sessions were attended by 30 participants, and all attendees chose to take part in the study. The face-to-face session had 14 participants (13 female, 1 male; three focus groups): eight medical educators, three pharmacy educators, two dentistry educators, and a medical education researcher. The online session had 16 participants (13 female, 3 male; three focus groups): five pharmacy educators, two medical educators, two pre-clinical sciences lecturers, two health professions’ education administrators, one physiotherapy educator, one nursing educator, one physicians’ associate educator, one psychology lecturer and one simulation educator.

### Researcher characteristics

All members of the research team have expertise across health professions’ education, workshop facilitation and qualitative methodologies. JM is a faculty developer with a research interest in uncertainty in health professions’ education. EAC is a faculty developer with expertise in professional identity. JH is a veterinary educator with a research interest in students’ management of uncertainty. SK is a clinical medical educator with a research interest in assessment methods. TP is an educationalist with expertise in clinical communication skills, role play-based training and several decades of experience as a principal investigator in health professions’ education studies.

### Data collection

Data were collected in a range of different formats. First, group artefacts, i.e., flip chart pages (face-to-face focus group) and shared presentation slides (online focus group), were captured, and text was extracted from these. Second, focus group discussions were audio-recorded (face-to-face focus group) or video-recorded (online focus group) and these were transcribed. Finally, field notes were kept by facilitators during the focus group discussions.

### Data analysis

Data from both study cohorts, face-to-face and online, were combined. Text from the focus group artefacts and from the transcribed discussions were organised using NVivo 12 (QSR International; Melbourne, Australia). Data were examined using a two-stage, hybrid approach of deductive and inductive thematic analysis based on a process proposed by Fereday and Muir-Cochrane [43]. In the first stage, JM created a coding framework based on questions that participants were asked to discuss in their focus groups which drew on Han and colleagues’ [29] taxonomy of uncertainty. These were:

- What sources of uncertainty exist here for Alice?
- What are the substantive issues of uncertainty that Alice faces here?
- What knowledge/skills/attitudes might help Alice manage uncertainty here?
- What sources of uncertainty exist here for Dena?
- What are the substantive issues of uncertainty that Dena faces here?
- What knowledge/skills/attitudes might help Dena manage uncertainty here?

Data were categorised by the researchers according to each question using themes and sub-themes. In the second stage, the data were examined again by JM using an inductive approach; this was carried out to screen for unexpected themes which may have been relevant to our research questions. The application of the coding framework to the dataset was discussed by JM, JH and EAC, and the findings of the analysis overall were discussed by all members of the research team.

## Results

Data analysis of the focus group interactions revealed four themes overall. Three themes related to categories that had been pre-determined by the coding framework: “Sources of uncertainty”, “Issues of uncertainty”, “Uncertainty attributes” (Table 2). One further theme was identified as a result of our second stage of inductive analysis: “Learning environment” (Table 3).

**Table 2:** Identified themes (deductive analysis)

	Locus of uncertainty: Alice		Locus of uncertainty: Dena
**Theme**	**Sub-theme**		
**Sources of uncertainty for the student**	Probability	• This patient interview may, or may not, have an impact on Alice’s academic progress • Alice may, or may not, learn from this situation	• Dena’s learning around the problem-based learning (PBL) topic may, or may not, be compromised • Dena may, or may not, miss out on an opportunity to learn from her colleagues in a team setting
	Ambiguity	• Alice isn’t able to fully engage with the patient meaning that the patient history is incomplete • There is a specific lack of details around psychosocial information concerning the patient • It is not clear whether or not the patient’s response is a symptom of a psychiatric disorder • There is a lack of trust in information related to consent, i.e., “Is this the right patient?”; “Is consent in place?” • Alice lacks clear options on how best to proceed, i.e., stay with patient or exit? which communication skills to use? • Alice lacks clear information on how she will be assessed on this interview	PBL is a teaching and learning strategy which naturally incorporates elements of ambiguity • There is a lack of induction to PBL and why it is used • There is a lack of instructions as to the specific task, e.g., details around goals, student roles, learning outcomes • There is a lack of clear guidance as to how assessment will take place
	Complexity	• The patient’s situation and presentation are inherently complex • Consent and capacity to consent are complex concepts • There appears to be a rapid change in the patient’s condition which culminates in withdrawal of consent • Alice experiences tension between two roles: a student who will be assessed *and* a healthcare professional, i.e., “How will this reflect on me?” versus “How am I to proceed in managing this patient?” • Alice’s apparent lack of experience and/or training adds difficulty to the situation	• PBL is an inherently complex teaching and learning strategy • The students are in their first year and lack experience with PBL • The environment, e.g., room set up, was not conducive to a successful PBL session • Key people, e.g., the facilitator and other group members, are absent from the session • Dena is balancing the priorities of different tasks, i.e., engaging with the PBL session or studying for an anatomy test
**Issues of uncertainty for the student**	Scientific	• Alice is not sure if this is the right patient • She is not sure if consent is in place • She cannot fully interpret the patient’s narrative or presentation • She is not sure how to approach the patient, or what to do or say; she is “bogged down in uncertainty” • She lacks clarity on whether to proceed with the interview or to exit • She struggles with how to apply her communication skills training in this particular setting • She is not sure how to change course when things don’t go to plan	• Dena is disoriented by the teaching approach overall • She lacks information about the benefits of PBL • She is not sure how to proceed with the task • She is not sure why she would engage with the task, or why it is important • She lacks clarity on how to gain reputable information to complete the task • She lacks a clear connection between this task and how it links back to the end goal of her education
	Practical	• She does not trust that the system around consent has been followed correctly • She not sure which role is most important within this educational setting, her role as a student or her role as a healthcare professional • She is unsure of the role of the healthcare team, and how to interact with them, in the care of this patient • She doubts the relevance or effectiveness of the communication training she has received to-date • She is not sure if, or how much, she should disclose about the situation to her tutor • She is unsure about how the assessment process works here	• She is unclear of the role of the facilitator and who is “in charge” • She is unclear of the role of the other group members and what to expect of them • She doesn’t know when, or if, the tutor is coming back • She doesn’t know her group, or where they are • She is unsure about how this group task will be assessed • She is unsure which task to prioritise: engage with the PBL session or study for the anatomy test • She lacks clarity about the attendance policy for this session, and she doesn’t know whether to stay or leave • Her efforts to engage with the task are hindered by lack of access to resources, e.g., effective Wi-Fi
	Personal	• She is not sure if she has the knowledge that she needs to handle this situation • She wonders why the communication skills that she has learned aren’t working for her • She has concerns around the consequences for her (“How will this reflect on me?”; “Am I going to get in trouble?”) • She has specific concerns around the consequences of this situation on her grades • She doubts herself with regards to how her approach with the patient (“Have I made a mistake?”) • She experiences upset, anger and/or frustration as a result of the situation	• She is not sure what her responsibilities are in this situation • She is confused as to why she’s not getting more support from the teaching staff • She feels a lack of trust in the facilitator and other group members • She is not sure if she wants to do this task; she experiences a lack of motivation • She experiences anxiety and a lack of confidence in her ability to retrieve solid information • She has concerns about how she is going to be marked • She has concerns about the fairness of the assessment process
**What knowledge/skills/ attitudes might help the student to manage this uncertainty?**	Knowledge	• More knowledge of this specific patient’s medical history • Boundaries (i.e., knowing when to stay and when to go, knowing what to put up with) • Help-seeking (i.e., knowing when/how to ask for help, knowing your team and who to call, knowing limits of capabilities) • Consent and capacity • The importance of the patient (i.e., knowing to place the patient at the centre of the learning) • The nature of uncertainty: “It’s not personal”	• More knowledge about PBL, its purpose and value • Uncertainty is part of the process in PBL • More knowledge about the specific session (e.g., the outcomes required, the specific assessment methods) • More knowledge about her classmates and the facilitator, and their roles and potential issues affecting them • More knowledge about the PBL topic and its importance • How group work takes place • How the session fits with the end goals of Dena’s profession • The nature of uncertainty: “It’s okay to not know”; “Sometimes the answers aren’t going to be perfect”; “Sometimes there is uncertainty and it’s just part of the process and you just have to go with a path”)
	Skills	• Communication skills (e.g., recognising psychosocial issues, attending to nonverbal skills, giving patients space to tell their story) • Managing difficult interactions • Empathy • Assertiveness • Emotional regulation (e.g., meditative strategies) • Self-awareness • Resilience • Reflective processes • Cooperation • Teamwork • Problem solving • Insight • Taking initiative	• Communication skills • Self-directed learning • Teamwork • Research skills • Problem solving skills • Information retrieval skills • Project management skills • Decision making despite incomplete knowledge
	Attitudes	• Openness • Adaptability/Capacity to change course • Collaborative attitude • Professionalism • Acceptance of patient difficulties • Showing an interest in learning • Growth mindset • Positivity • Confidence/Experience	• Openness to new ideas and processes • Motivation • Respect for others • Value learning from others • Growth mindset • Positive outlook

**Table 3:** Identified theme (inductive analysis)

		Locus of uncertainty: Alice	Locus of uncertainty: Dena
**Theme**	Sub-theme		
**Learning environment**	Reducing uncertainty	• Providing more information around assessment and how marking will happen in the context of a difficult situation	• Having trained faculty, moderating process for assessments • Providing expert high quality content and resources; having a third party proofread instructions • Provide preparatory session about the nature of PBL and the need to develop this skill • Have teaching staff experience a PBL session themselves • Nurture trust in the environment
	Role of the educator	• Importance of educators as role models in challenging situations • Tolerant student/supervisor relationships	• Calibrating expectations between staff and students
	Evidence-based teaching strategies	• More practice of difficult patient scenarios/awkward conversations • Simulation as an approach to prepare students and develop these skills • Debriefing and exploring the student’s uncertainty, i.e. where the challenge arose, why they chose their course of action? • Learning opportunities which integrate communication and teamwork	• Knowing the importance of buy-in – explain to students why PBL is used • Appealing to students’ values or personal drivers • Thinking about the group dynamics
	Addressing the culture around uncertainty	• Normalising uncertainty for students	• Rewarding students for engaging with uncertainty • Signposting to students that managing uncertainty is part of maturing as a health professional

### Sources of uncertainty

Using Han *et al*.’s taxonomy of uncertainty as a guide, focus group participants discussed many different sources of uncertainty for the students in the role played scenarios. These sources were categorized by the researchers into sub-themes which reflected the taxonomy: probability, ambiguity and complexity.

For example, participants noted how the future indeterminacy of events, or probability, could provide a source of uncertainty for the student. In the case of the clinical student, Alice, the participants talked about the different ways in which the situation could unfold for her:

“*She does express an uncertainty about her grading and how she will be perceived based on that interaction although it wasn't clear unless the woman complained, she probably wouldn't get into trouble.*” [Medical education researcher, female]

Participants also shared ideas about how a lack of clear or trustworthy information (ambiguity) contributed to uncertainty in both scenarios:

*“There seems to be an ambiguity in terms of the grading process as well, so that might be another source.”* [Medical educator, male]

*“They had been given a lecture, I suppose, but they don't really know where to find the information or are unsure where to find the information.”* [Medical educator, female]

Discussions also highlighted aspects of the situations which made it harder for each student to understand (i.e., complexity). For example, participants picked up on the tension experienced by Alice in balancing her identity as a medical student with that of a new healthcare professional:

*“So for the student, the greater consequence is ‘How will this reflect on me?’ as opposed to ‘What are the issues for the patient?’ So the student has the dilemma of having to fulfill both roles, one, in how they're performing and being assessed, and, two, as a… as a physician, even in their early days, ‘How am I to proceed in managing this patient?’”* [Medical educator, female]

Finally, there were occasional instances where participants expressed confusion as to what aspects of the students’ experiences could be classified as sources or issues.

*“OK. So where would… that fall? Would that fall in issues or sources?”* [Focus group facilitator]

*“No idea. I'm very uncertain around this.”* [laughing] [Health professions’ education administrator, female]

### Issues of uncertainty

Our participants were also able to identify different issues of uncertainty for each student which were organised by the researchers into sub-themes according to Han *et al*.’s taxonomy [29]: scientific, practical and personal.

With regards to scientific, or data-centred, issues, participants observed how the situation had led to specific knowledge gaps for the students around their learning experience:

*“She wasn't sure whether she should sit, she should sit on the chair to the… she should stand up closer to the patient on the bed? So she didn't know how to approach this patient.”* [Clinical educator, female]

*“It's their first encounter maybe with PBL so… very disorientating for people who are used to conventional, didactic teaching. Very disorientating.”* [Medical educator, male]

There were multiple comments which related to how the system around the student, or practical issues, represented specific issues of uncertainty:

*“And it wasn't until later on she started questioning was the patient consented, is this the right patient? Surely there should have been a process there to make sure all of that had happened at the outset?* [Pharmacy educator, female]

*“… they were given a task but no tools on how to do it. They weren't given the tools.”* [Medical educator, female]

Finally, participants were able to discern different ways in which the impact of these uncertainties could be experienced by students from a personal perspective. Personal issues of uncertainty that were mentioned included:

*“You can see that in this situation her self-doubt was really kicking in.”* [Pre-clinical sciences lecturer, female]

*“It was something about her lack of understanding kind of made her, again, uncertain of how to proceed or what to even look at. That was… a source of anxiety for her.”* [Simulation educator, female]

### Uncertainty attributes – role-play specific

Our third theme, uncertainty attributes, stemmed from a section in the session where participants were asked to discuss what knowledge, skills and attitudes may have helped students to manage uncertainty within the context of the specific role play (Table 2).

With regards to knowledge, participants highlighted that more background knowledge of the topic at hand, e.g. a better patient history for Alice or a better understanding of MRSA for Dena, could have helped the students. They also listed specific types of knowledge for each student. For example, they considered that Alice could have benefited from more knowledge around setting boundaries and knowing when and how to ask for help, whilst Dena could have benefited from more knowledge about PBL, its purpose and value.

The participants also commented that the students might find it useful to have more insight around the nature of uncertainty in health professions’ education:

*“Sometimes you just have to go with a path.”* [Physician’s associate educator, female]

*“It's okay to not know…”* [Pre-clinical sciences lecturer, female]

Participants listed numerous skills that could have helped these students. Notably, communication, teamwork and problem-solving skills appeared relevant to both Alice and Dena. Similarly, many attitudes were discussed with openness, growth mindset and positivity/positive outlook observed across both lists.

### Learning environment

The final theme that was identified was “Learning environment.” This theme covered a range of different comments which addressed participants’ perspectives on how the learning environment could play a role in the uncertainty experienced by these students, or other students in similar situations. The comments were categorised according to four sub-themes: “Reducing uncertainty”; “Role of the educator”; “Evidence-based teaching strategies”; and, “Addressing the culture around uncertainty.”

The first sub-theme focused on the ways that uncertainty in the learning environment can be reduced for students:

“*If you're running a course, it's very important to make sure that all the guidance documents and the resources are available and arranged and provided in a logical order. So maybe it's a good idea sometimes to get somebody who's not directly involved to proofread everything, you know, as a fresh eye just so that, um, things are optimal for the students coming in*.” [Health professions’ education administrator, male]

The next sub-theme highlighted the importance of the educator and educator-student relationships in helping students to navigate such uncertain situations.

*“I suppose that maybe the tutor is available or it might be that they feel they can report back uncertainties. You know… as educators we say "if you've any problems come and tell me sooner rather than me hearing about it at the end of the week"... do you know?"* [Medical educator, female]

*“It's important to have role models where they actually see somebody dealing with this difficult situation. It's all very well to talk about it in a room, but to see a skilled physician like [focus group participant] dealing with it...”* [Medical educator, male]

One participant highlighted how the taxonomy would help her to be more open to students’ experience of uncertainty:

“*When I first saw it without understanding the framework, I was just thinking, good God, she's so negative. It was just a judgment. Whereas actually now that I'm understanding more about uncertainty, it probably was a vulnerability on her part rather than the negativity. And that probably happens with a lot with students*.” [Pharmacy educator, female]

The participants also outlined how evidence-based teaching strategies could have helped the students to feel more prepared for the situations they faced. For example, greater opportunities to practise and get feedback on challenging situations was mentioned:

*“I suppose just what might help Alice… more practice, I suppose on those kinds of scenarios in the simulation environment I think first. If… if she hasn't got a chance to do that, you know, somewhere safe?”* [Physician’s associate educator, female]

A final sub-theme addressed how the culture around uncertainty in health professions’ education could be acknowledged and explored with students.

*“You know, if that could somehow be rewarded or reflected or captured or somehow… Like reward uncertainty, if that makes sense? Like say, ‘Uncertainty is a good thing. You should have it, you should share it. We should work on it. It's an important skill.’"* [Pharmacy educator, female]

### Attributes

Finally, our participants were asked to contribute to a list of attributes, knowledge, skills and attitudes, which they considered would provide a foundation to undergraduate health professions’ students in managing uncertain situations. These included an awareness of the nature of uncertainty within healthcare practice (i.e., “health professions’ work has many grey areas as opposed to black/white ones”; “uncertainty is not always bad”), an ability to recognise uncertainty, and adopting attitudes of adaptability, positivity, and resilience. A full list of proposed uncertainty management attributes is presented in Table 4.

**Table 4:** Knowledge, skills and attitudes which support undergraduate health professions’ students to manage uncertain situations

Knowledge	
• Core medical knowledge (e.g., consent topics)	
• How to define boundaries	
• How and when to escalate care or call for help	
• Knowing what to do when you don’t know what to do	
• The purpose and value of teaching strategies (i.e., “priming” for learning)	
• An awareness of others’ issues and roles (e.g., other classmates and patients)	
• The centrality of the patient in healthcare	
• The nature of uncertainty within healthcare practice (i.e., “health professions’ work has many grey areas as opposed to black/white ones”; “uncertainty is not always bad”)	
• Dunning-Kruger effect	
Skills	
• Recognising uncertainty	
• Communication skills	
• Managing challenging situations	
• Emotion regulation	
• Self-assessment	
• Self-directed learning	
• Working with feedback	
• Reflective practice (e.g., journaling)	
• Assertiveness	
• Taking initiative	
• Teamwork skills	
• Problem solving	
• Research skills	
• Information retrieval skills	
• Project management skills	
• Decision making despite incomplete knowledge	
• Ethical decision making	
Attitude	
• Openness	
• Adaptability	
• Motivation	
• Value learning from others	
• Growth mindset	
• Positivity	
• Self-awareness	
• Collaborative attitude	
• Tolerance	
• Resilience	
• Engagement	
• Trust	
• Confidence/Experience	

## Discussion

Revisiting our research question, we reflect on the ways in which Han *et al*.’s taxonomy can support health professions educators’ to better recognise and conceptualise uncertainty. The findings from this study indicate that this taxonomy of uncertainty can be pragmatically applied to a faculty development setting. Specifically, the framework allowed participants to achieve a greater depth of understanding around students’ experiences of uncertainty than they might have achieved had they worked without it.

Using the taxonomy, participants were able to identify multiple sources and issues of uncertainty for the students, thus unveiling the extent of uncertainties encountered by health professions’ learners in relatively commonplace circumstances. Whilst it may seem paradoxical to want to unpack an experience of uncertainty into further, multiple uncertainties, this can be an important opening step to managing such situations. As noted by Han *et al*. [29], “uncertainty is not a monolithic phenomenon. There are multiple varieties of uncertainty, which may have distinct psychological effects and thus warrant different courses of action.” By recognising and teasing out the separate sources and issues, educators can identify, or help students to identify, more adaptive responses to uncertainty.

It was also considered that the taxonomy worked well in the focus group setting. Previous studies that have used Han *et al*.’s taxonomy to organise experiences of uncertainty have tended to describe one-to-one approaches such as in-depth interviews [31, 34, 35]. To the authors’ knowledge, this is the first time that the taxonomy was used in a group setting. The level of detail provided by the participants around the students’ uncertainties was surprisingly high and suggests that using the taxonomy in a group setting, where individuals hold different personal responses and perspectives, can uncover a more complete range of sources and issues of uncertainty. Perhaps this process, beyond the taxonomy itself, stimulated reflection on what constitutes ‘good’ management of uncertainty or, indeed, ‘good’ teaching around management of uncertainty?

This was also the first time, to our knowledge, that the taxonomy was applied to simulated rather than real-life experiences. We chose this approach as we considered it a low-risk way of introducing educators to student uncertainties. In practice, this took the form of a perspective-taking exercise, where educators were asked to focus on the students in the vignettes. There was some evidence that this approach helped educators to develop more open approaches to student uncertainty; one participant described a greater empathy towards one of our role play students as a result of engaging with the taxonomy. This is an important faculty development issue, considering the salience of the educator-student relationships in mitigating the uncertainties that students encounter in the course of their studies.

Overall, we consider that this framework provides a valuable tool for faculty to approach conversations around uncertain situations. As Han *et al.* [33] explain, the taxonomy has an important function in “promoting shared awareness of otherwise unconsidered sources and issues of uncertainty, and enabling stakeholders to approach these uncertainties in an organized manner.” In the context of education, this taxonomy can provide an entry point and guiding framework, promoting dialogue around uncertainty that might otherwise be avoided. The taxonomy could also be used to help educators and students engage in shared reflection around the specific sources and issues of uncertainty in any given situation, facilitating shared mental models and improved decision-making processes.

Whilst most of our data related to the taxonomy and activities used in the intervention, our analysis also revealed a theme around how the learning environment contributed to the uncertainty experienced by these students. Despite being asked to focus on the knowledge, skills and attitudes that could help these students, the discussion often drifted towards what the participants might have done differently in organising teaching. The issues highlighted were: reducing uncertainty for the students, the role of the educator, using evidence-based teaching practices, and addressing the culture around uncertainty. This digression from the task at hand may have resulted from the choice of role plays; in both cases there was a perception that students had been let down by some level of failure in their education. Alternatively, this might have represented participants’ efforts to prevent uncertainty from arising in teaching, rather than acknowledging that students will likely experience uncertainty as part of their learning experience.

Our study revealed that participants, when faced with this in-depth and extensive appreciation of uncertainty within the scenarios, tended to respond in different ways. There were responses that were oriented towards students’ learning and development (e.g., using these to make explicit the nature of uncertainty to students, and defining the skills to cope with such situations). However, there were also responses that were oriented towards reducing or removing the uncertainty for the student, with many suggestions as to how to “fix” the situations that Alice and Dena found themselves in. A drive to reduce uncertainty has been well documented within the health professions’ practice. As Han *et al.* [45], say: “Physicians and other health care providers manage these effects and their experience of uncertainty itself through various strategies, but principal among these is the effort to seek information to reduce uncertainty. Nearly every major clinical activity that physicians undertake—diagnostic, prognostic, and therapeutic—is part of this overarching effort.” However, our findings here suggest that that this drive to reduce uncertainty extends beyond clinical activities into those of teaching.

Whilst reducing uncertainty for students and patients is important, it may not always be possible, or, indeed, the right course of action. The value of identifying these uncertainties is not so much that they can be prevented by preparing the student, but more that they can be used as focal points for reflection and discussion, supporting the student to manage, respond to and cope with uncertainty. In this study, participants did seem to recognise that there are times for educators to step in and times to step back. For example, it may be appropriate to reduce uncertainty for students at specific points in their training e.g., to support them as they enter clinical rotations or during first exposure to problem-based learning, with a gradual ‘stepping back’ of this supervisory management of the environment, being replaced by exposure to uncertainty. It’s likely that such a scaffolded approach to uncertainty can be approached practically through shared reflective strategies where students and tutors employ tools such as the taxonomy used here [46].

Within the theme of learning environment we also noticed the appearance of a more positive, accepting narrative around the culture of uncertainty. Past research has highlighted that uncertainty can be viewed from a negative perspective by educators and students [14]. Here, participants commented that uncertainty should be highlighted as normal and “a good thing”, and that development of skills to manage uncertainty can be viewed as part of the maturation process within undergraduate health professions’ education. This may suggest that faculty development interventions, such as the one described here, can promote a ‘stop and reflect’ approach for educators, i.e., encouraging them to pause to consider, and communicate to students, the importance of productive uncertainty in the learning process.

Our second research question enquired as to what health professions’ educators considered as key attributes for undergraduate learners with respect to managing uncertainty. When asked to consider the knowledge, skills and attitudes that could help our role play students, and undergraduate health professions’ learners more generally, to manage uncertainty, the participants listed multiple and diverse attributes. Here we will focus on the more general attributes (Table 4) as these were deemed of greatest relevance to our research question.

Perhaps somewhat unsurprisingly, our participants considered that having a firm foundation in core medical knowledge would help reduce uncertainty for health professions’ learners. They also commented that it would be helpful for students to have a greater understanding of their role, as well as that of their colleagues and the patient, in healthcare settings. Some comments related to the nature of uncertainty and how it would benefit students to understand that uncertainty is “normal” and “not always bad” in healthcare. Other additions to the list, i.e., “knowing what to do when you don’t know what to do”, served to reinforce how even experienced educators have difficult pinning down specific, observable steps to help students in managing uncertainty.

With regards to skills that may help students to manage uncertain situations, these included communication, emotional regulation, problem solving, information management, ethical decision making and an ability to self-assess. Each of these skills are supported within the existing health professions’ literature [14]. Again, there were additions to the list, e.g., “taking the initiative”, that were more vague and harder to define. One addition to the list, “recognising uncertainty” was of particular interest. Whilst we know that health professions’ learners meet uncertainty in multiple stages of their training, it’s likely that many learning opportunities are missed. The literature signposts that experiences of uncertainty commonly happen “under the radar” in healthcare settings, e.g., Mackintosh & Armstrong [47] highlight that “uncertainty work… may not be directly experienced or categorised as such by those undertaking it.” This leads us to ask if it may be helpful to prime both educators and students to notice when uncertainty emerges in the course of learning. It may be that using a “lens” of uncertainty to explore difficult situations could be a useful approach in the context of shared educator-student or student-student reflections.

Finally, attitudes thought to be helpful in managing uncertainty were: openness, adaptability, positivity, and a growth mindset. Again, these have been supported in the literature [48–50]. Although student attitudes are typically viewed as more difficult to expand on, such ‘intangibles’ can be developed experientially and through structured reflection, e.g., through small group ‘debrief’ tutorials or personal journals.

### Strengths and limitations

A key strength of this study is the successful application of Han *et al.*’s taxonomy [29] to a faculty development session, opening the door to further explorations of its use within educational contexts. We consider that the taxonomy is a practical and versatile framework which can help to empower health professions’ educators when working with students to manage uncertain situations derived from real-life experiences. Whilst this study uses the taxonomy to focus on the perspective of a student (i.e., the student as the locus of uncertainty), our intervention could be easily adapted to explore the uncertainties faced by educators and clinicians from a wide range of socio-cultural and professional backgrounds. We also consider that overall instructional design of this session could be adapted to the context of undergraduate health professions’ education, providing a valuable opportunity for students to diagnose and classify aspects of uncertainty that they meet during their training.

Although there were occasions where the participants expressed confusion with regards to the sources and issues of uncertainty, these were not frequent, and did not appear to detract from the utility of the tool overall. On this basis, we recommend Han *et al.*’s taxonomy as a conceptual framework of interest, which has potential application in a wide variety of uncertainty management educational interventions.

The study also resulted in a list of knowledge, skills and attitudes which can be used in discussions around how to manage uncertainty in the context of health professions’ education. Our list of attributes, whilst not exhaustive, can be used by educators and students alike in considering how best to work with, or respond to, an uncertain situation.

There were, however, several limitations associated with this study. Our study sample size was small, and, due to the opt-in nature of both faculty development sessions, it’s likely that participants held some pre-existing interest in uncertainty management. Further to this, almost all participants were based at health professions’ education institutions in Ireland. Individuals’ approaches to uncertainty can be heavily influenced by professional and socio-cultural variables [51–53], and, thus, our findings may not be representative of the views and experiences of a more diverse faculty cohort.

In addition, offering the intervention as part of a conference setting added time restrictions to its design. Participants were presented with just two role-played scenarios which aimed to balance depth of enquiry with feasibility. More time, and a greater number and diversity of scenarios, may have yielded different results. Finally, we believe that any future delivery of a similar intervention should include the active participation of health professions’ students. Our focus in this study was the development of faculty and our intervention design used a perspective-taking approach, i.e., our participants were asked to consider uncertainty from the viewpoint of a student. Whilst we consider this approach was a valid way to provoke thought and discussion around uncertainty attributes, it would have helped to have health professions’ students take part in the facilitation of the session to bring real-world depth to our role-played scenarios.

## Conclusions

This study highlights the successful use of Han *et al.*’s taxonomy of uncertainty within a faculty development setting. The taxonomy provides a effective conceptual framework which educators can use to identify a wide range of sources and issues of uncertainty for students within simulated scenarios. Our findings suggest that Han *et al*.’s taxonomy is a practical and versatile tool in designing faculty development interventions around uncertainty management. Participants in this study also contributed to a list of uncertainty attributes, i.e. knowledge, skills, and attitudes, which may be useful to undergraduate health professions’ learners in managing uncertainty. Finally, our findings suggest that health professions’ educators can sometimes feel compelled to reduce or remove uncertainty for students, which may not always be the most appropriate course of action. We propose that more faculty development in this domain is likely to be required.

## Data Availability

The datasets analysed during the current study are available from the corresponding author on reasonable request.
